# Dietary Strategies for Gut Barrier Integrity in Inflammatory Bowel Disease: The Impact of Fiber and Beyond

**DOI:** 10.7759/cureus.101355

**Published:** 2026-01-12

**Authors:** Bindiya Verma, Khaleel Ahmed Manik, Sachin Verma, Amita Kumari, Anita Kumari, Mohammed Jaffer Pinjar

**Affiliations:** 1 Physiology, Faculty of Medicine and Health Sciences, Integral Institute of Medical Sciences and Research, Integral University, Lucknow, IND; 2 Pediatric Intensive Care Unit, Vivekananda Polyclinic and Institute of Medical Sciences, Lucknow, IND; 3 Physiology, All India Institute of Medical Sciences, Deoghar, IND

**Keywords:** dietary fiber, gut microbiota, inflammatory bowel disease, nutrition management, personalized diet

## Abstract

Dietary fiber is increasingly viewed as a promising adjunct in inflammatory bowel disease (IBD) management due to its potential to support gut barrier function, shape the intestinal microbiota, and influence inflammatory pathways, yet many patients restrict fiber during flares and remission to reduce symptoms despite uncertainty about the long-term consequences of such avoidance. This narrative review synthesizes mechanistic, clinical, and real-world evidence on dietary fiber characteristics, tolerability, and associations with IBD outcomes, with attention to how fiber quality and intake context vary across patient groups. Overall, available findings suggest that adequate, appropriately selected fiber may contribute to gut homeostasis and symptom control, but substantial gaps persist regarding the most suitable fiber types, effective amounts, and practical food sources that balance benefit with tolerability. Although guidance increasingly favors maintaining adequate fiber intake when feasible, more rigorous, patient-centered intervention studies and improved characterization of real-world intake patterns are needed to enable personalized recommendations and safe integration of fiber-rich foods into individualized care.

## Introduction and background

Over the past decade, research on the role of dietary fiber (DF) in gut health and inflammatory bowel disease (IBD) has expanded considerably. Growing data indicate that DF can enhance overall well-being, ease IBD symptoms, regulate inflammatory activity, and positively reshape the gut microbiome [1-5]. In line with these findings, recent international guidelines now recommend adequate fiber intake during the remission phase of both Crohn’s disease (CD) and ulcerative colitis (UC) [6-8]. Moreover, the traditional view that patients with stricturing CD should avoid DF is shifting toward the inclusion of soluble fibers provided in adapted textures.

Despite these advances, actual fiber consumption among individuals with IBD remains suboptimal. Compared with both national DF guidelines and intake levels observed in healthy populations, IBD patients consistently report lower fiber consumption [9-10]. This discrepancy highlights the gap between current evidence-based recommendations and real-world dietary practices.

Currently, healthcare professionals providing nutritional guidance to patients with IBD encounter a significant knowledge gap regarding the optimal types and amounts of DF that can be both well tolerated and clinically beneficial across the active and remission phases of the disease [11,12]. Furthermore, growing evidence indicates that fiber tolerance, as well as its metabolic degradation into short-chain fatty acids (SCFAs), is highly dependent on the composition and function of the host microbiome. This interdependence underscores the need for a more personalized, microbiome-informed nutritional approach to optimize fiber intake in individuals with IBD [13,14].

Although multiple dietary patterns have been proposed for individuals with IBD, such as the plant-based diets, Mediterranean diet, anti-inflammatory diets, and specific carbohydrate diet, they vary substantially in how they advise using or avoiding fiber-rich foods. Despite these differences, only a small number of intervention trials in adults with IBD have directly evaluated the effects of whole food-derived DF on disease activity and on associated changes in the gut microbiome.

This narrative review outlines the prevailing nutritional guidance on DF intake in individuals with IBD. This review aims to delineate the dietary models most commonly investigated in the context of IBD across various clinical settings, to identify the unmet clinical needs related to interventions using natural fiber sources rather than fiber supplements, and to examine existing knowledge gaps and prospective research directions. This review places particular focus on the practical challenges of quantifying intake of fiber-rich foods in everyday settings and on approaches to support sufficient consumption among people living with IBD.

## Review

Current perspectives on DF

DFs are generally described as carbohydrate polymers resistant to digestion, which are fully or partially fermented in the large intestine [15]. Recent developments in nutritional science and policy have led to an expansion of this definition. Across several regions, including Canada, Europe, New Zealand, Australia, China, and Brazil, dietary guidelines now recognize non-digestible carbohydrate polymers comprising either 10 or more and three to nine monomeric units [16]. This expanded classification reflects an evolving understanding of the physiological roles of DFs and their contribution to human health.

Classification and functional characteristics of DF

DFs are commonly classified by their chemical composition, botanical origin, and physicochemical properties [17]. Fermentability is one of the key physicochemical properties and is primarily governed by structural determinants such as degree of polymerization, linkage pattern, and structural accessibility to microbial enzymes; solubility and viscosity are related traits that often correlate with, but do not alone determine, fermentative potential [18,19]. Furthermore, this process is dynamically modulated by the composition, diversity, and metabolic activity of the intestinal microbiota [20,21]. The fermentation of DFs yields SCFAs, which are widely recognized for their beneficial physiological effects, particularly in supporting gastrointestinal health [22].

Prebiotic properties of DF

Although all DFs, including insoluble components such as cellulose and lignin, are generally subject to varying degrees of bacterial fermentation, the designation of a fiber as prebiotic is more specific. According to the International Scientific Association for Probiotics and Prebiotics (ISAPP), a prebiotic is defined as “a substrate that is selectively utilized by host microorganisms conferring a health benefit” [23]. In this context, prebiotics serve as selective nutrient substrates for a limited range of microbial genera or species, most notably Bifidobacterium and Lactobacillus, which possess the enzymatic capacity to metabolize these compounds. This targeted fermentation enhances the growth of beneficial bacteria and promotes the production of SCFAs, thereby contributing to host health [24].

The concept of microbiota-accessible carbohydrates (MACs) has been introduced to address the limitations of earlier fiber classifications. MACs are defined as dietary carbohydrates that escape enzymatic digestion and absorption by the host, thereby becoming metabolically available to the gut microbiota. Unlike traditional categorizations, this terminology emphasizes the functional interaction between dietary substrates and microbial communities. Dietary MACs can originate from a variety of sources, including plants, animal tissues, and microbial components present in foods. Regardless of their origin, their defining characteristic is their requirement for microbial metabolism within the gut. This broader framework also encompasses substrates traditionally categorized as prebiotic fibers, reflecting their role in sustaining beneficial microbial activity [25].

**Figure 1:**
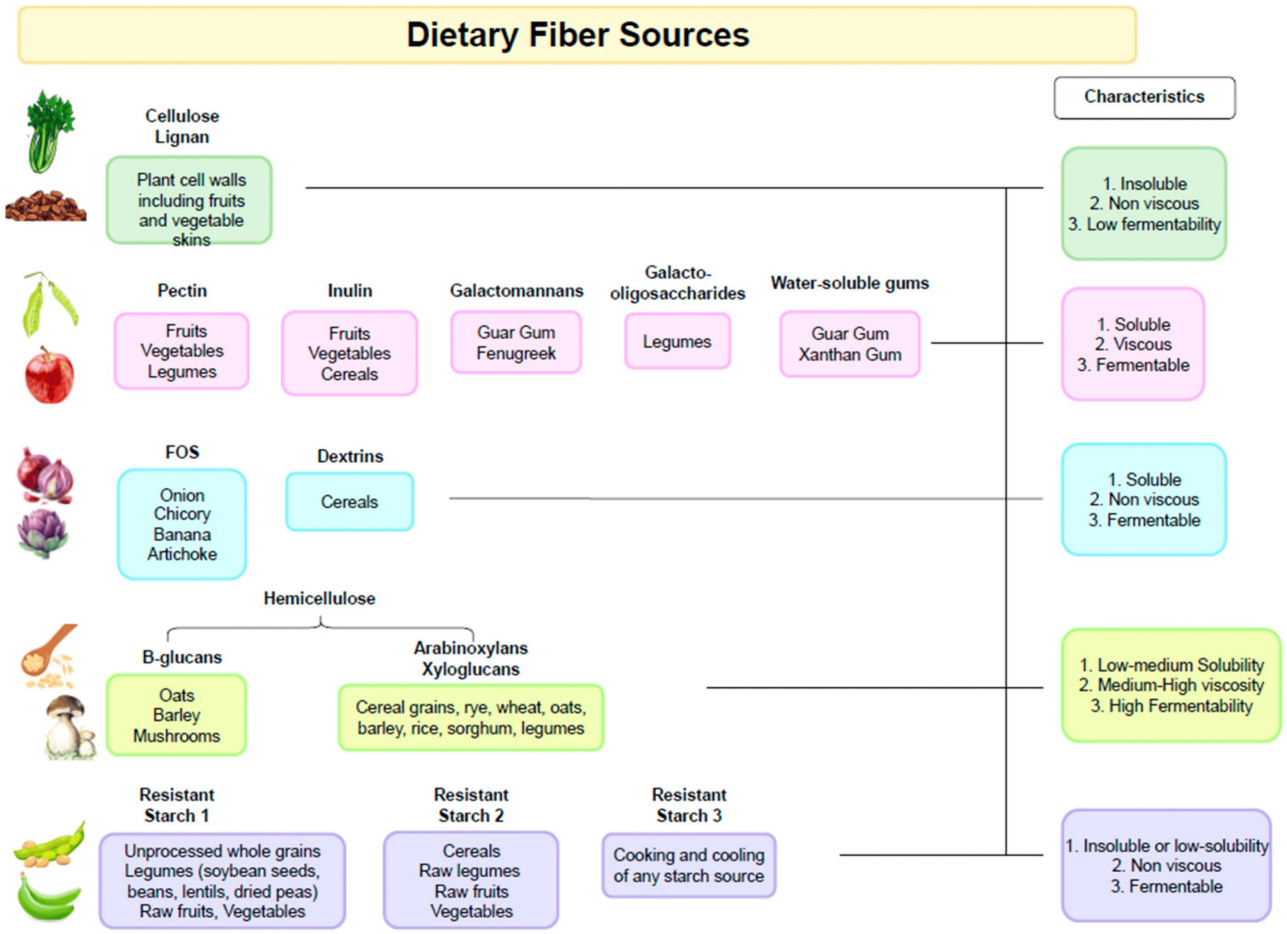
Dietary fiber sources. Open access journal under a CC-BY license Contributed by Loy L, Petronio L, Marcozzi G, et al.: Dietary Fiber in Inflammatory Bowel Disease: Are We Ready to Change the Paradigm?​ [5]​​​​​​

It is important to recognize that the fibers derived from different plant sources exhibit considerable variation in their chemical composition and physicochemical properties (Figure 1) [5].

For instance, pectin occurs in higher concentrations in fruits and certain vegetables, whereas β-glucans are predominantly present in cereals. Similarly, resistant starch is found in notable amounts in pulses, cereals, and potatoes. Accordingly, dietary patterns rich in diverse plant-derived foods, each supplying unique forms of fiber, can influence multiple biological functions within the digestive system and promote the establishment of a more heterogeneous gut microbial ecosystem [26,27].

Suboptimal fiber intake in adults with IBD: patient-reported insights

The consumption of DFs among adults with IBD remains markedly below recommended levels relative to both individuals without the disease and prevailing dietary standards. A recent comprehensive review analyzing 26 studies involving 4,164 individuals with IBD assessed dietary sufficiency in relation to national nutrition benchmarks and intake patterns of healthy counterparts. The analysis revealed that participants with IBD consistently exhibited lower total fiber consumption than control groups, with average intakes remaining below recommended national levels regardless of disease status [10].

Multiple factors contribute to this reduced fiber intake among patients with IBD. These include dietary beliefs, adoption of self-directed elimination diets, guidance from healthcare professionals, and information obtained through digital or non-specialist sources [28]. One prevalent patient perception is that diet serves as a potential trigger for IBD relapse. Consequently, fiber-rich food groups, such as raw fruits, vegetables, particularly cruciferous varieties, and legumes, are often minimized or excluded, despite specialist recommendations not supporting such restrictions [29-32].

The situation is further complicated by the high prevalence of overlapping functional symptoms. Approximately 40% of patients with IBD in remission also meet criteria for concomitant irritable bowel syndrome (IBS) [33]. In an attempt to manage symptoms such as bloating, abdominal pain, and altered bowel habits, many adopt a low-FODMAP (fermentable oligo-, di-, and monosaccharides and polyols) dietary pattern. However, such dietary modifications are frequently implemented without professional dietetic supervision, often leading to unnecessary and overly restrictive food choices [34,35]. This practice results in further reductions in fiber consumption, particularly of prebiotic short-chain oligosaccharides, which play a key role in gut microbial health.

Variability and risks in dietary recommendations for patients with IBD

Dietary guidance provided to patients with IBD is frequently inconsistent and, at times, contradictory. This variability carries the risk of unnecessary and prolonged dietary exclusions, as well as potentially harmful nutritional practices. Health professionals, including gastroenterologists, IBD nurse specialists, and dietitians, are the primary sources of nutritional counselling for this population [31,36-38]. However, there remains a substantial risk that patients may receive dietary guidance inconsistent with current evidence-based standards regarding fiber consumption.

A substantial proportion of patients with IBD continue to be advised to adopt low-fiber or so-called *low-residue *diets, typically characterized by fiber intakes of approximately 10 g/day [39,40]. Such recommendations stem from historical practices in which low-residue diets were prescribed for the temporary control of gastrointestinal discomfort and to minimize fecal volume [41,42]. While effective in select acute settings, this approach has often been extended indiscriminately. In many cases, patients are not adequately counselled on the need for gradual reintroduction of DF following symptom resolution or clinical remission [43]. Consequently, restrictive practices may be prolonged unnecessarily, exacerbating already inadequate fiber consumption and raising concerns over long-term nutritional adequacy and gut health.

Misinformation and the role of nutritional education in fiber intake

The Internet represents a widely used but potentially unreliable source of dietary information for patients with IBD. As illustrated by Hou et al., an analysis of 32 websites providing nutritional advice revealed that 72% recommended avoidance of high-fiber diets or fiber-rich foods [28]. Such online content may perpetuate misconceptions, contributing to restrictive eating patterns and reinforcing unwarranted fears regarding the role of fiber in symptom management and disease relapse.

Importantly, when offering dietary guidance, health professionals must also consider that insufficient fiber consumption remains prevalent even in the general population, with habitual intakes frequently falling below national recommendations [44-47]. Thus, nutritional interventions in individuals with IBD should not only focus on counteracting disease-related dietary restrictions but also address the broader context of low fiber consumption observed across the population. This highlights the necessity for structured, evidence-based dietary education as a core component of IBD management, ensuring patients receive accurate, consistent guidance that supports both symptom control and long-term nutritional adequacy.

Nutritional recommendations in IBD regarding fiber intake

In recent years, growing insights into DFs' contribution to gastrointestinal function have highlighted the need to maintain sufficient fiber intake in individuals with IBD [48]. Although human evidence concerning the impact of fiber on IBD prevention and treatment is still comparatively scarce, clinical interventions have shown favorable effects [49]. These include improvement of gastrointestinal symptoms, enhancement of health-related quality of life, modulation of systemic and intestinal inflammation, regulation of immune responses, and mitigation of gut microbial dysbiosis by supporting restoration of the microbiome [50-52]. These positive effects are largely mediated through the fermentation of MACs, leading to the production of SCFAs [53-55], as well as through preservation of the intestinal mucus layer by promoting mucus synthesis and secretion [17,56].

In response to the growing recognition of these benefits, international and national organizations have sought to create evidence-based nutritional guidelines for patients with IBD, tailored to disease phenotype and activity. Notably, the International Organization for the Study of Inflammatory Bowel Diseases (IOIBD) [6], the European Society for Clinical Nutrition and Metabolism (ESPEN) guidelines on Clinical Nutrition in IBD (2023) [7], and the British Dietetic Association (BDA) consensus statement (2022) [8] provide consistent recommendations aligned with those for the general population, emphasizing adequate consumption of fiber through plant-based foods.

While randomized controlled trials (RCTs) do not currently endorse any specific whole-food dietary intervention as primary therapy for inducing or maintaining remission in active disease, nutritional recommendations broadly encourage patients with IBD to include adequate amounts of vegetables, fruits, and complex carbohydrates, while limiting intake of refined sugars. Exceptions may be warranted in individuals with concurrent functional bowel disorders, such as IBS, coexisting with CD or UC when active inflammation is not present. In these instances, a temporary low-FODMAP diet may be considered [57-60], but only under appropriate dietetic supervision. This approach should be implemented for limited periods, followed by gradual and personalized reintroduction of FODMAP-containing foods according to individual tolerance. Such a strategy is essential to balance symptomatic relief with the prevention of long-term nutritional inadequacy and micronutrient deficiencies.

Exceptions to fiber recommendations in IBD

An important exception to the general recommendation for adequate DF intake applies to individuals with symptomatic or clinically significant stricturing CD. In such cases, personalized nutritional support may be warranted, particularly with respect to limiting the insoluble fraction of DF while emphasizing soluble fiber sources, accompanied by adequate hydration. This tailored approach aims to reduce the risk of symptom exacerbation while maintaining the potential benefits of fiber intake.

The ESPEN practical guidelines on Clinical Nutrition in IBD highlight the role of food consistency in patients with symptomatic small bowel strictures. Specifically, the guidelines recommend adapting food texture by incorporating tender, well-cooked, and skin-free vegetables, along with blended or skin-removed fruits often prepared as smoothies to enhance tolerance and minimize the risk of mechanical obstruction [7].

Nonetheless, although limiting insoluble fiber may be appropriate for patients exhibiting symptoms of stricturing CD, existing evidence does not endorse routine fiber avoidance in individuals without active symptoms. A recent comprehensive review determined that there is insufficient rationale for widespread DF restriction among patients with CD lacking obstructive manifestations [60]. Although a definitive consensus has yet to be reached regarding the optimal type, quantity, and preparation of fiber in IBD diets, there is growing professional agreement that blanket recommendations to avoid DF are unwarranted. Instead, clinical practice is gradually shifting toward personalized, evidence-based strategies that promote safe consumption of fiber to support overall gastrointestinal and metabolic health.

DFs consumption in IBD dietary patterns

Multiple clinical investigations have examined the effects of DF interventions in adults with IBD, encompassing both active disease states and periods of remission. However, the available body of evidence remains limited, as well-designed interventional and observational studies addressing DF intake in the context of whole foods are scarce. Current clinical and pre-clinical research protocols have increasingly shifted toward the evaluation of isolated fiber types or fiber extracts, which do not adequately capture the complexity of habitual diets or the diversity of DF-rich foods.

In patients with CD without a stricturing phenotype, various nutritional interventions incorporating fiber-rich foods have been investigated. Chiba et al. [61] and Levenstein et al. [62] designed dietary regimens encouraging the consumption of legumes, whole grains, fruits, and vegetables, and compared these to either an omnivorous diet (OD) or a low-residue diet. In the study by Levenstein et al., no statistically significant differences were observed between groups with respect to symptomatology, nutritional status, hospitalization, surgical requirements, new complications, or postoperative recurrence [62]. By contrast, Chiba et al. reported that patients adhering to a semi-vegetarian diet (SVD) achieved significantly higher rates of clinical remission and exhibited lower relapse rates at both one-year and two-year follow-up compared with those consuming an OD [61].

In dietary intervention studies, patients with CD have demonstrated similar tolerance to increased fruit and vegetable intake, even when the intervention cohort showed no significant difference from the control group in overall consumption, as both were advised to enhance intake. Notably, this intake level exceeding the national mean was well accepted by patients throughout both active disease and remission periods, alongside their routine therapeutic regimens [63].

Similar outcomes were reported in a multicenter RCT by Lewis et al., where individuals with CD exhibiting mild to moderate activity adhered to either a Mediterranean diet (MD) or a specific carbohydrate diet (SCD) [64]. Both dietary patterns emphasized fresh fruits and vegetables, and participants successfully increased their intake of DF relative to baseline levels. The trial reported no significant differences between groups with respect to symptomatic remission, clinical remission, or reductions in inflammatory biomarkers. However, adherence to the MD was greater compared to the SCD, suggesting a potential practical advantage of the MD in long-term disease management [64].

Further evidence was provided by Brotherton et al., who investigated the impact of DF enrichment on gastrointestinal outcomes in patients with CD in stable remission. Participants were randomized to receive a structured dietary intervention consisting of one-half cup of wheat bran cereal daily, alongside general guidance to reduce sugar intake, or to a control group instructed to avoid whole grains, dairy products, and spicy foods. Over a four-week RCT, the high-DF and low-refined carbohydrate intervention was found to be both feasible and well tolerated, with no adverse effects reported. Notably, this approach was associated with improvements in gastrointestinal function and quality of life among participants [51].

Limitations, recommendations, and future directions

The available evidence on DF in IBD remains limited by heterogeneous study designs, short follow-up durations, and variability in the characterization of fiber type, dose, and food sources. These constraints hinder direct comparison across trials and reduce the generalizability of findings to diverse IBD phenotypes and real-world dietary patterns.​

Future research should prioritize well-designed RCTs that evaluate whole-food, fiber-rich dietary patterns in clearly defined IBD subgroups, including patients with active disease, stricturing phenotypes, and overlapping functional bowel symptoms. Studies should incorporate standardized methods to quantify fiber intake and quality, integrate microbiome and metabolite profiling, and use clinically meaningful endpoints such as remission rates, quality of life, and need for hospitalization or surgery.​

Clinicians and dietitians are encouraged to move away from blanket low-fiber recommendations and instead adopt individualized, stepwise strategies to increase fiber intake using tolerable plant-based foods, while monitoring symptoms and nutritional status. Structured dietary education, alignment of advice with contemporary guidelines, and close collaboration between gastroenterologists, dietitians, and patients are essential to safely translate emerging evidence into routine practice.

## Conclusions

The understanding of DF in IBD has shifted from routine restriction toward cautious, individualized inclusion as part of comprehensive care. Current evidence indicates that appropriately selected and gradually introduced fiber can support gut barrier function, foster a more favorable microbiota, and improve gastrointestinal symptoms and quality of life, particularly in patients in remission. At the same time, rigid low-fiber practices remain common, often driven by outdated guidance, patient fears, and inconsistent information from non-specialist sources.

To bridge the gap between evolving science and everyday practice, nutritional strategies for IBD should emphasize personalized assessment of disease phenotype, symptom profile, and dietary preferences. Rather than universal avoidance, patients should be supported to reintroduce and diversify fiber-rich foods in forms and amounts that they can tolerate, with clear education and ongoing monitoring. A coordinated, evidence-informed approach has the potential to improve both disease-related outcomes and overall nutritional well-being in this population.
